# Comparison of Postoperative Chronic Groin Pain After Repair of Inguinal Hernia Using Nonabsorbable Versus Absorbable Sutures for Mesh Fixation

**DOI:** 10.7759/cureus.35562

**Published:** 2023-02-27

**Authors:** Puneet K Agarwal, Tarun Sutrave, Devashish Kaushal, Raghvendra Vidua, Rajesh Malik, Ajeet P Maurya

**Affiliations:** 1 General Surgery, All India Institute of Medical Sciences, Bhopal, IND; 2 Urology, All India Institute of Medical Sciences, Bhopal, IND; 3 Forensic Medicine, All India Institute of Medical Sciences, Bhopal, IND; 4 Radiodiagnosis, All India Institute of Medical Sciences, Bhopal, IND

**Keywords:** suture, mesh fixation, absorbable versus nonabsorbable, inguinal hernia, chronic groin pain

## Abstract

Background: Inguinal hernia repair is one of the most common operations performed in general surgery. Lichtenstein mesh hernioplasty is a commonly practiced technique for open inguinal hernia repair. Out of many other complications postoperatively, chronic groin pain is one of the patients' most common postoperative complaints. There is no direct evidence to explain the cause of post-mesh hernioplasty pain. Only a few studies have been done to judge the effect of suture material used for mesh fixation on chronic groin pain.

Aims and objectives: To compare the postoperative groin pain level in mesh hernioplasty using nonabsorbable versus absorbable sutures for mesh fixation at predetermined intervals using a visual analog scale (VAS) score.

Methods: A prospective, single-center, non-randomized, observational study was conducted. All patients per inclusion and exclusion criteria of inguinal hernia planned for surgery were admitted electively on the day of surgery and were operated on in minor OT under local anesthesia for open mesh hernioplasty. The VAS score assessed the postoperative pain level.

Results: This observational study was done to look for any difference in postoperative chronic groin pain after mesh fixation with either nonabsorbable, prolene sutures (PS) or absorbable vicryl sutures (VS). One hundred and ten patients fulfilling the department of general surgery inclusion criteria were admitted to the study. In our study, postoperatively, the incidence of chronic groin pain was assessed and followed up to six months. After six months, 25%of patients had pain. Of this 25%, the majority (70%) of patients had mild pain, 15% had moderate pain, and 15% had severe pain. There was no statistically significant difference between the two groups of mesh fixation by nonabsorbable versus absorbable sutures.

Conclusion: Inguinal hernia is one of the most typical conditions seen in general surgery clinics with male predominance. Definitive management of inguinal hernia is surgery. There is no difference in postoperative chronic groin pain with either type of suture material i.e., nonabsorbable or absorbable (prolene vs vicryl) sutures. To conclude, fixation material for mesh does not influence chronic inguinodynia. However, further studies are required for the same.

## Introduction

Inguinal hernia repair is a frequently performed operation in general surgery. And the Lichtenstein mesh hernioplasty is a commonly practiced technique for open inguinal hernia repair [1]. Among many other postoperative complications, chronic groin pain is one of the most common postoperative complaints. It persists even after repair, and this complication significantly impacts the quality of life [2]. This chronic post-mesh hernioplasty pain occurs in 16% to 62 % of patients who have undergone inguinal mesh hernioplasty [3]. There is no direct evidence to explain the cause of post-mesh hernioplasty pain. But, possibly, nerve damage during surgery may be one of the causes [4]. Few studies have been done to judge the effect of suture material used for mesh fixation on chronic groin pain. But there is ample evidence that nonabsorbable alloplastic meshes and suture made of polypropylene or polyester excite inflammatory foreign body reaction that progresses to chronic inflammation, fibrosis, dense adhesion to the surrounding, nerve entrapment, and pain [5].

Since many studies have yet to be taken to study the prevalence of inguinal hernia in developing countries, the assumption is that hernia affects 15% to 20% of the general population; the prevalence of inguinal hernia in India is estimated to be 1.5 to 2 million [5]. Inguinal hernia is most common in men than women. About 90% of inguinal hernia repairs are done in men, whereas 70% of femoral hernia repairs are performed in women. The estimated lifetime risk of inguinal hernia in men is 27% and 3% in women [6]. The prevalence of inguinal hernia is age dependent, and in males, it has a bimodal distribution curve with the first peak in the first year of age and the second peak after the fourth decade of life [7]. Though femoral hernia is most common in women, overall, inguinal hernias are five times more common than femoral hernias. The most common subtype of groin hernia in men and women is the indirect inguinal hernia. The ratio between indirect hernia and direct hernia in men is 2:1 [8]. 

In this study, we observed the effect of the suture material used for mesh fixation on postoperative chronic groin pain.

## Materials and methods

Aims and objectives

Here, we compare the postoperative groin pain level in mesh hernioplasty using nonabsorbable versus absorbable sutures for mesh fixation at predetermined intervals using a visual analog scale (VAS) score. This is a prospective, single-center, non-randomized, observational study that was conducted after seeking permission from the institutional human ethics committee of All India Institute of Medical Sciences, Bhopal, India (approval no. IHEC-LOP/2019/MD0053) for the duration of the follow-up, which is a minimum of six months from the date of surgery. The proposed sample size was calculated to obtain a power of 80% with a confidence interval of 95%. Based on this, fifty-one is required in each of these two groups. Thus, a total of 110 patients with 55 in each group were recruited for the study.

Inclusion & exclusion criteria

The sampling method used was purposive sampling. All patients ranging from 18 to 65 years who planned for elective inguinal hernia repair were included in the study. Patients with recurrent inguinal hernia, incarcerated hernia, physical or mental disorders that could interfere with the evaluation of pain scores, impaired cognition, those on daily analgesics for any other illness, those that refused to consent to participate in the study, and those with a history of surgery in the groin area were excluded from the study.

Study procedure

All patients with inguinal hernias visiting our institute (All India Institute of Medical Sciences, Bhopal, India) were thoroughly examined in the outpatient department. A complete history was taken, followed by a clinical examination of the patient, and findings were methodically noted.

Preoperatively, patients were assessed for groin pain on a numerical rating scale of 1 to 10 using the visual analog scale (VAS). Pain gradation into four categories depending on the VAS scores as follows: nil=VAS score 0, mild=VAS score 1-3, moderate=VAS score 4-6, severe=VAS score >6. Patients using analgesics preoperatively for pain were also noted.

After taking informed consent, patients who were planned for surgery were admitted electively on the day of surgery and were operated on in the minor operation theatre under local anesthesia.

## Results

This observational study looked for any difference in postoperative chronic groin pain after mesh fixation with either a nonabsorbable prolene suture (PS) or an absorbable vicryl suture (VS). As per the inclusion and exclusion criteria, 110 patients were recruited for the study from the department of general surgery. After thorough clinical evaluation and investigations, all the patients were operated on, and all of them underwent surgery under local anesthesia. Intraoperatively, the type of suture used to fix the mesh was noted down. Patients of all ages as per inclusion and exclusion criteria were included in the study.

Age distribution

**Figure 1:**
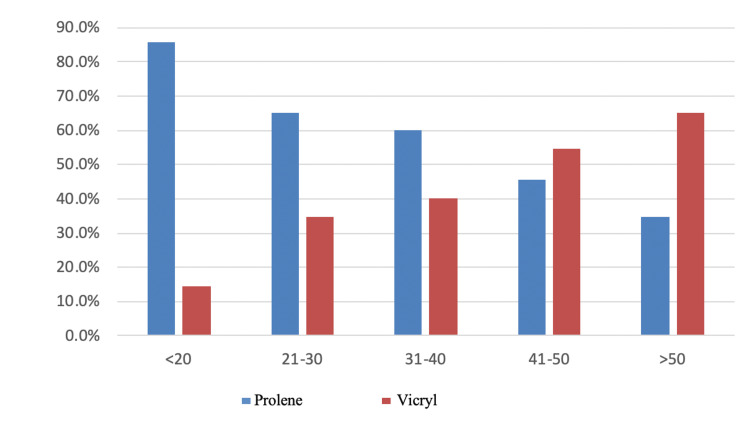
Double bar graph showing the age at presentation in the two groups

In our study, 20.9% of patients were between 21 to 30 years, 20% were between 41 and 50 years, and 39% were >50 years. As per the inclusion criteria age, those aged less than 18 years and more than 65 years were not included in the study. Hence the classic bimodal distribution of hernia patients wasn’t obtained. The mean age was 44±15 years. The age of patients ranged from 18 to 67 years. The mean age in the PS group was 40+15 years and in the VS group was 48+14 yrs. The equality of means (t-test) was calculated for these two groups and was found to have a significant difference in age distribution between the groups. This difference might be due to the non-randomization of the study. Table 1 and the Figure 1 double bar graph show the age at presentation.

**Table 1:** Age at presentation PS: Prolene suture, VS: Vicryl suture, SD: Standard deviation

Age at presentation		N	Mean in years	SD	p-value
	PS Group	55	40.20	15.996	0.008
	VS Group	55	48.16	14.733	

Sex ratio

**Figure 2:**
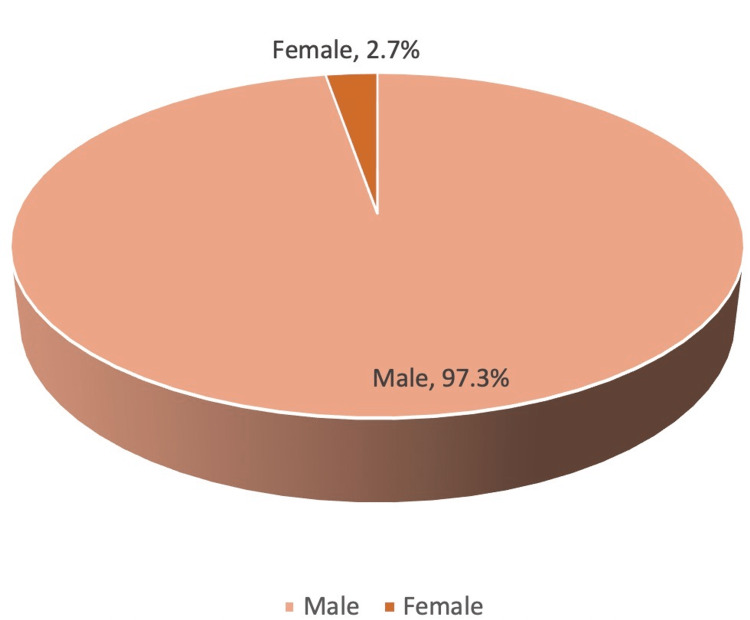
Pie chart for sex ratio

**Figure 3:**
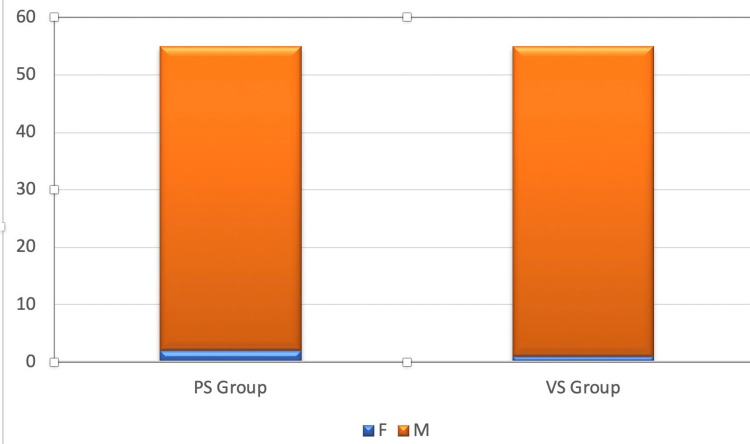
Bar graph showing the sex ratio in both groups PS: Prolene suture, VS: Vicryl suture, F: Female, M: Male

In this study, 97% of patients were males, and 3% were females. The male:female ratio was 32:1. Table 2, the Figure 2 pie chart, and the Figure 3 bar chart represent the sex ratio.

**Table 2:** The sex ratio in the two groups

			Group		Total	p-value
			Proline	Vicryl		
sex	Female	Count	2	1	3	
		Percentage % within the sex	66.7%	33.3%	100.0%	
	Male	Count	53	54	107	
		Percentage % within the sex	49.5%	50.5%	100.0%	
Total		Count	55	55	110	
		Percentage % within the sex	50.0%	50.0%	100.0%	0.558

Preoperative pain VAS score was mild in 51% of patients and of moderate nature in 49% of patients. The distribution of such patients in both groups was not statistically significant as tested by the Pearson chi-square test (Tables 3, 4 represent preoperative pain scores).

**Table 3:** Preoperative pain score VAS: Visual analog scale

Preoperative pain score (VAS)	Number of patients	Percentage
Mild	20	51.28%
Moderate	19	48.71%
Total	39	100%

**Table 4:** Preoperative pain VAS score grading in each group VAS: Visual analog scale

				Group		Total	p-value
				Prolene	Vicryl		
Preoperative pain score (VAS)	Mild		Count	11	9	20	
			% within mild pain score (VAS)	55.0%	45.0%	100.0%	
	Mod		Count	10	9	19	
			% within moderate pain score (VAS)	52.6%	47.4%	100.0%	
Total		Count		21	18	39	
		%		53.8%	46.2%	100.0%	0.882

Preoperative use of analgesics

In this series, 9% of patients with hernias were on painkillers due to pain associated with swelling. Tables 5, 6 show the preoperative use of analgesics.

**Table 5:** Percentage of patients with preoperative use of analgesics

Symptoms	Number of patients	Percentage
Patients with hernia	110	100%
Pain with swelling	39	35.45%
Preoperative use of analgesics	10	9.09%
Hernia patients free of analgesics	100	90.90%

**Table 6:** Percentage of patients with preoperative use of analgesics in each group

			Group		Total	p-value
			Prolene	Vicryl		
Preoperative use of analgesia	NO	Count	17	12	29	
		% not within use of analgesics	58.6%	41.4%	100.0%	
	Yes	Count	4	6	10	
		% within preoperative use of analgesics	40.0%	60.0%	100.0%	
Total		Count	21	18	39	0.308

**Figure 4:**
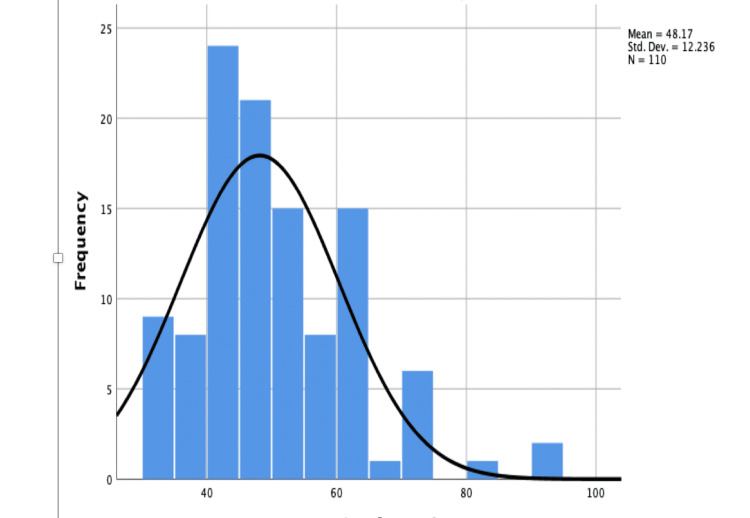
Distribution of surgery in each group Std.Dev: Standard deviation

The operative time was defined as the time from administering regional block anesthesia to the end of the surgical procedure, and operative time ranged from 30 minutes to 90 minutes. The mean operative time in the PS group was 44 minutes, and in VS group was 52 minutes. The difference was found statistically significant after calculating the p-value. The Figure 4 distribution curve and Table 7 represent the duration of surgery.

**Table 7:** Mean of the duration of operation in each group SD: Standard deviation

Group		N	Mean duration (in minutes)	SD	p-value
Operative time	Prolene	55	44.38	9.024	0.001
	Vicryl	55	51.96	13.839	

Postoperative groin pain

Pain VAS Score After 24 Hours

**Figure 5:**
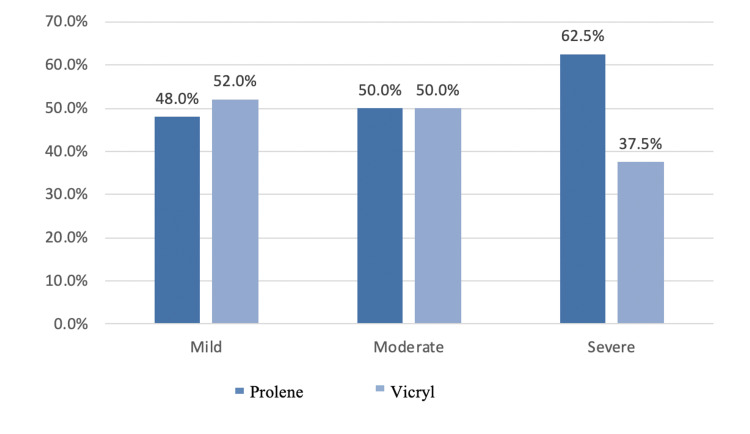
Bar graph showing 24-hour postoperative pain VAS score in both groups VAS: Visual analog scale

In this series, almost all patients had pain during postoperative day one after hernia repair. Around 46% had mild pain, 47% had moderate pain, and 7% had severe pain. There was no statistically significant difference between the two groups. Table 8 and the Figure 5 bar graph represent postoperative pain after 24 hours.

**Table 8:** The 24-hour postoperative pain VAS score VAS: Visual analog scale

			Group		Total	p-value
			Prolene	Vicryl		
Pain VAS score at 24 hours	Mild	Count	24	26	50	
		Percentage within the mild group	48.0%	52.0%	100.0%	
	Moderate	Count	26	26	52	
		Percentage within the moderate group	50.0%	50.0%	100.0%	
	Severe	Count	5	3	8	
		Percentage within the severe group	62.5%	37.5%	100.0%	
Total		Count	55	55	110	0.748
		Percentage	50.0%	50.0%	100.0%	

Pain VAS Score After Seven Days

**Figure 6:**
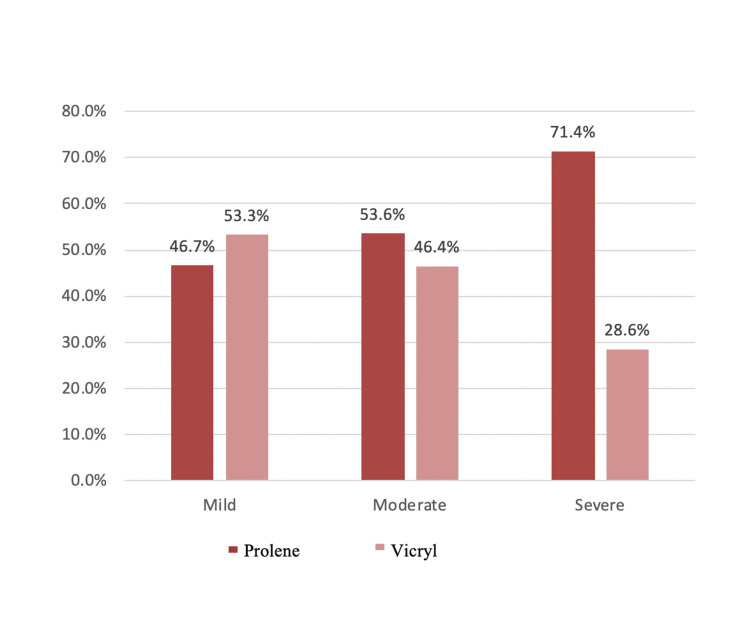
Bar graph showing seventh-day postoperative pain VAS score in both groups VAS: Visual analog scale

In this series, during postoperative day seven after hernia repair, almost all patients had pain. Around 68.2 % had mild pain, 25.45% had moderate pain, and 6.3% had severe pain. Further distribution of patients in respective groups in each category has been shown in Table 9. There was no statistically significant difference between the two groups. Table 9 and the Figure 6 bar graph represent pain after seven days of surgery.

**Table 9:** Seventh-day postoperative pain VAS score VAS: Visual analog scale

			Group		Total	p-value
			Prolene	Vicryl		
Pain VAS score after seven days	Mild	Count	35	40	75	
		% within the mild group	46.7%	53.3%	100.0%	
	Moderate	Count	15	13	28	
		% within the moderate group	53.6%	46.4%	100.0%	
	Severe	Count	5	2	7	
		% within the severe group	71.4%	28.6%	100.0%	
Total		Count	55	55	110	0.414

Pain VAS Score at One Month

**Figure 7:**
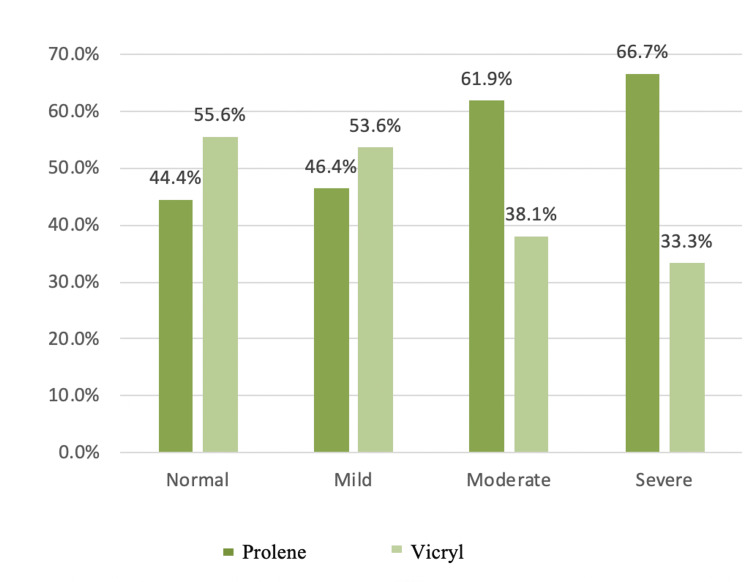
Bar graph showing one-month postoperative pain VAS score in both groups VAS: Visual analog scale

In this series, one month postoperatively after hernia repair, 25% were completely free of pain, 51% had mild pain, 19% had moderate pain, and 5% had severe pain. Further distribution of patients in respective groups in each category has been shown in Table 10. There was no statistically significant difference between the two groups. Table 10 and Figure 7 show the pain VAS scores one month after the surgery.

**Table 10:** Postoperative pain VAS score in both groups one month after surgery VAS: Visual analog scale

Pain VAS score at 1 month	Normal	Count	12	15	27	
		% within the normal category	44.4%	55.6%	100.0%	
	Mild	Count	26	30	56	
		% within the mild category	46.4%	53.6%	100.0%	
	Moderate	Count	13	8	21	
		% within the moderate category	61.9%	38.1%	100.0%	
	Severe	Count	4	2	6	
		% within the severe category	66.7%	33.3%	100.0%	
Total		Count	55	55	110	0.480

Pain VAS Score at Six Months

**Figure 8:**
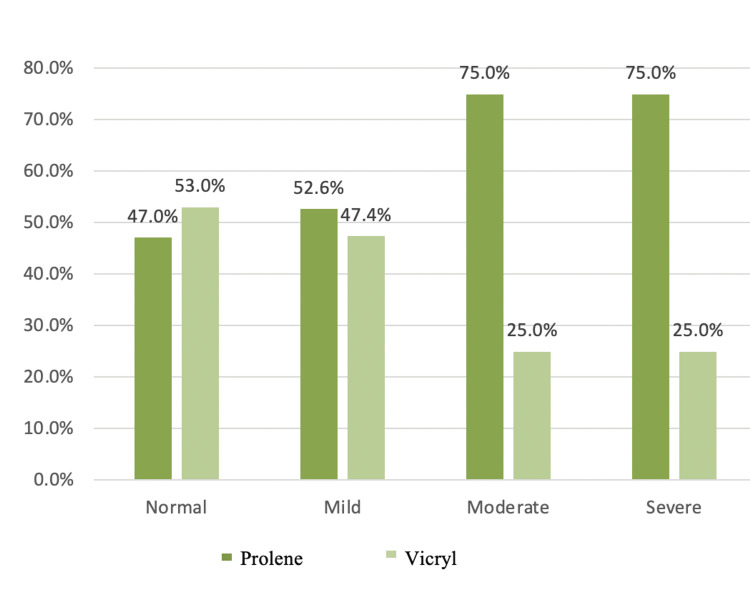
Bar graph showing the six-month follow-up of postoperative pain VAS scores in both groups VAS: Visual analog scale

In this series, during the six-month follow-ups, the percentage of people with chronic groin pain (defined as pain existing for over three months) after hernia repair was 25%. Around 75% were completely free of pain, 17% had mild pain, 4% had moderate pain, and 4% had severe pain. Further distribution of patients in respective groups in each category has been shown in the table. There was no statistically significant difference between the two groups. Table 11 and the Figure 8 bar graph show the six-month postoperative score.

**Table 11:** Six-month postoperative pain VAS score in both groups VAS: Visual analog scale

			Group		Total	p-value
			Prolene	Vicryl		
Pain VAS score at six months	Normal	Count	39	44	83	
		% within normal	47.0%	53.0%	100.0%	
	Mild	Count	10	9	19	
		% within mild pain	52.6%	47.4%	100.0%	
	Moderate	Count	3	1	4	
		% within moderate pain	75.0%	25.0%	100.0%	
	Severe	Count	3	1	4	
		% within severe pain	75.0%	25.0%	100.0%	
Total		Count	55	55	110	0.502
			50.0%	50.0%	100.0%	

Pain at Rest at Six Months

In this series, 8% of patients had pain at rest during the six-month follow-up (Table 12).

**Table 12:** Percentage of patients with pain at rest in both groups

			Group		Total	p-value
			Prolne	Vicryl		
Pain at rest	No	Count	49	52	101	
		% within this category	48.5%	51.5%	100.0%	
	Yes	Count	6	3	9	
		% within this category	66.7%	33.3%	100.0%	
Total		Count	55	55	110	0.297
		%	50.0%	50.0%	100.0%	

Pain During an Activity at Six Months

**Figure 9:**
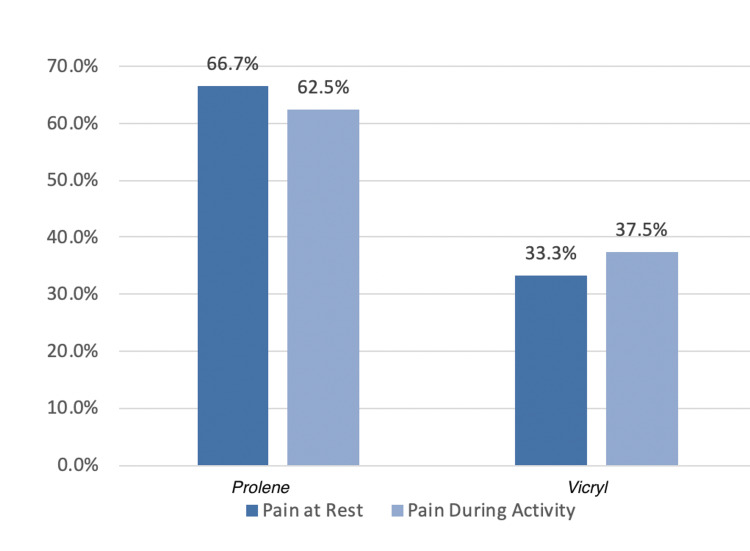
Bar graph showing pain at rest, and pain during an activity in both groups

Both groups had no difference in pain during an activity at six months. Overall, 7% of patients had pain during an activity at six months. Table 13 depicts pain during an activity in both groups. Figure 9 is a bar graph depicting pain at rest and during activity in both groups after six months.

**Table 13:** Percentage of patients with pain during an activity in both groups

			Group		Total	p-value
			Prolene	Vicryl		
Pain during activity	No	Count	48	52	100	
		% within this category	48.0%	52.0%	100%	
	Yes	Count	5	3	8	
		% within this category	62.5%	37.5%	100%	
Total		Count	53	55	108	0.541
		%	49.1%	50.9%	100% of patients with pain during an activity in both groups	

Mean of Pain VAS Score and p-value

On calculating each group's VAS score means and the p-value (Mann Whitney U test), the p-value was insignificant for 24 hours, seven days, and six months. For one month, the p-value was significant, but Pearson's chi-square of mild, moderate, and severe categories at one month was insignificant. The p-value at six months was also insignificant. So, this significance doesn't have much importance (Table 14).

**Table 14:** Mean of pain VAS scores and p-values VAS: Visual analog scale

	Prolene group	Vicryl group	Prolene group	Vicryl group	Prolene group	Vicryl group	Prolene group	Vicryl group
	Pain score (VAS) at 24 hours	Pain score (VAS) at 24 hours	Pain score (VAS) on the 7th day	Pain score (VAS) on the 7th day	Pain score (VAS) at 1 month	Pain score (VAS) at 1 month	Pain score (VAS) at the 6th month	Pain score (VAS) at the 6th month
Mean	4.05	3.87	3.29	2.73	2.73	1.91	1.09	0.55
Median	4.00	4.00	3.00	2.00	3.00	2.00	0.0	0.0
Std. Deviation	1.693	1.441	1.792	1.407	2.086	1.713	2.048	1.425
Minimum	1	2	1	1	0	0	0	0
Maximum	8	8	8	7	7	7	7	7
p-value	0.559264		0.120985		0.0380714		0.193984	

Pain VAS Score at Six Months and Preoperative Pain Relationship

On cross-tabulation between pain VAS score at six months and preoperative pain, there was no significant association or relationship in the Pearson chi-square test (Table 15).

**Table 15:** Pain VAS score at six months after surgery and preoperative pain relationship VAS: Visual analog scale

			Preoperative pain score VAS			Total	p-value
			Nil	Mild	Mod		
Pain VAS at six months	Normal	Count	59	14	10	83	
		% within pain VAS score for six months	71.1%	16.9%	12.0%	100.0%	
	Mild	Count	8	4	7	19	
		% within pain VAS score for six months	42.1%	21.1%	36.8%	100.0%	
	Moderate	Count	3	1	0	4	
		% within pain VAS score for six months	75.0%	25.0%	0.0%	100.0%	
	Severe	Count	1	1	2	4	
		% within pain VAS score for six months	25.0%	25.0%	50.0%	100.0%	
Total		Count	71	20	19	110	0.059

## Discussion

Chronic inguinodynia is a groin pain that develops post-hernia repair and lasts more than three months. It can be classified as nociceptive and neuropathic pain. Nociceptive pain is either somatic or visceral in origin and is due to the periosteal reaction, scar tissue, and mechanical pressure caused by folded mesh called 'meshoma'. The cause of neuropathic pain is unintended entrapment of nerve in suture or mesh, perineural fibrosis, partial division, crushing of nerve, or diathermy burns, all leading to chronic nerve irritation [9]. Of these two mechanisms, neuropathic pain is the leading cause of postoperative inguinodynia [10]. Hidalgo et al. studied mesh suture fixation vs glue fixation and concluded that glue fixation had less incidence of chronic groin pain when compared to suture fixation [11].

There have been studies comparing glue vs absorbable sutures for mesh fixation in Lichtenstein hernioplasty. A randomized control trial by Paajanen et al. concluded that rates of foreign body sensation, acute and chronic pain, and recurrence rates were similar in both absorbable and nonabsorbable groups [12]. Jeroukhimov et al. conducted a single-blind randomized clinical trial comparing the effect of absorbable braided sutures (vicryl) and nonabsorbable monofilament sutures (prolene) on the rate of chronic pain in inguinal hernia repair and the results showed that nonabsorbable suture is associated with a higher rate of chronic pain and took a long time for pain disappearance as compared with absorbable sutures [4]. Ismail et al. in a systematic review and meta-analysis compared sutureless repair and self-gripping sutures and did not favor either of the two fixation techniques in aspects of chronic groin pain and recurrence [13].

In our study, postoperatively, the incidence of chronic groin pain after six months was 25%. Of this 25%, around 70% had mild pain, 15% had moderate pain, and 15% had severe pain. In 25% of the patients with groin pain, 13.6 % were on analgesics, 3.6% needed painkillers daily for pain management, and 10% required analgesics sometimes only. The incidence of postoperative chronic groin pain after six months was statistically insignificant in both groups. Therefore in our study, the fixation of mesh with either absorbable or nonabsorbable sutures doesn’t influence the incidence of chronic groin pain.

This was also concluded in the study by Paajanen et al., where it was studied if absorbable sutures caused less pain than continuous polypropylene fixation of the mesh in the Lichtenstein operation. Study results concluded that absorbable suture material does not appear to cause less neuropathic pain than nonabsorbable sutures [12]. In a contrary study by Meena et al., it was concluded that absorbable sutures for mesh fixation had less groin pain as compared to nonabsorbable sutures in hernia repair, with 155 participants in each group [14].

To conclude, further randomized studies with larger sample sizes and longer follow-ups are required to provide level 1 evidence. Also, studies depicting the pathophysiological mechanisms causing chronic groin pain and interactions between the mesh materials and host tissue are suggested, which might help to understand chronic groin pain better. Also, there is a need for mesh material that balances both recurrence and chronic groin pain.

## Conclusions

Inguinal hernia is one of the most prevalent conditions seen in general surgery clinics, predominantly in males. The definitive management of inguinal hernia is surgery. Fixation material for mesh does not influence chronic inguinodynia and recurrence rates. Further randomized studies with larger sample sizes and longer follow-ups are required to provide level 1 evidence in this regard. Also, studies depicting the pathophysiological mechanisms causing chronic groin pain and interactions between the mesh materials and host tissue are suggested for a better understanding of chronic groin pain. The surgery for hernia repair has evolved with many changes, modifications, and concepts to decrease the incidence of recurrence.
